# The Bulk of Autotaxin Activity Is Dispensable for Adult Mouse Life

**DOI:** 10.1371/journal.pone.0143083

**Published:** 2015-11-16

**Authors:** Aggeliki Katsifa, Eleanna Kaffe, Nefeli Nikolaidou-Katsaridou, Aris N. Economides, Susan Newbigging, Colin McKerlie, Vassilis Aidinis

**Affiliations:** 1 Division of Immunology, Biomedical Sciences Research Center Alexander Fleming, Athens, Greece; 2 Genome Engineering Technologies Group and Skeletal Diseases TFA Group, Regeneron Pharmaceuticals Inc., Tarrytown, New York, United States of America; 3 Physiology and Experimental Medicine Research Program, the Hospital for Sick Children, Center for Phenogenomics, Toronto, Canada; 4 Department of Laboratory Medicine and Pathobiology, Faculty of Medicine, University of Toronto, Toronto, Canada; The University of Tennessee Health Science Center, UNITED STATES

## Abstract

Autotaxin (ATX, *Enpp2*) is a secreted lysophospholipase D catalysing the production of lysophosphatidic acid, a pleiotropic growth factor-like lysophospholipid. Increased ATX expression has been detected in a number of chronic inflammatory diseases and different types of cancer, while genetic interventions have proven a role for ATX in disease pathogenesis. Therefore, ATX has emerged as a potential drug target and a large number of ATX inhibitors have been developed exhibiting promising therapeutic potential. However, the embryonic lethality of ATX null mice and the ubiquitous expression of ATX and LPA receptors in adult life question the suitability of ATX as a drug target. Here we show that inducible, ubiquitous genetic deletion of ATX in adult mice, as well as long-term potent pharmacologic inhibition, are well tolerated, alleviating potential toxicity concerns of ATX therapeutic targeting.

## Introduction

Autotaxin (ATX, *Enpp2*) is a secreted lysophospholipase D [1] widely present in biological fluids including blood [2–4]. ATX is the major enzyme catalysing the production of lysophosphatidic acid (LPA) and ATX heterozygous null mice have half of normal LPA levels [5–7]. LPA is a bioactive, growth factor-like lysophospholipid promoting a large variety of cellular responses in almost all cell types [2, 8], mediated from at least six G-protein coupled LPA receptors (LPARs) that exhibit overlapping specificities and widespread distribution [9, 10].

ATX expression and LPA production were shown to be necessary for embryonic development, as ubiquitous genetic deletion of ATX resulted to vascular and neuronal defects leading to lethality [5–7]. In adult life ATX is widely expressed, and upregulated levels have been reported in various chronic inflammatory diseases and cancer [2, 11, 12]. Conditional genetic deletion of ATX attenuated the development of rheumatoid arthritis and pulmonary fibrosis in mouse models [13, 14], suggesting a primary role in disease pathogenesis [15, 16]. Transgenic mammary ATX overexpression resulted in enhanced rates of spontaneous breast cancer development in aged mice, often preceded by chronic inflammation [17]. Therefore, ATX overexpression at chronic inflamed or neoplastic tissues, possibly localized to the cell surface through integrin-binding [18], most likely results to increased local levels of LPA, which in turn exert its pathologic effects at the microenvironment [2].

Consequently, ATX has emerged as a promising therapeutic target in chronic inflammatory diseases and cancer [2] and a large number of ATX inhibitors have been developed [19, 20]. Moreover, the crystal structure of ATX was recently solved [18, 21] enabling rational drug design. In this report we show that inducible, ubiquitous genetic deletion of ATX in adult mice, as well as its potent pharmacologic inhibition, are well tolerated, alleviating potential toxicity concerns of ATX therapeutic targeting.

## Materials and Methods

### Mice

All mice were bred at the animal facilities of the Alexander Fleming Biomedical Sciences Research Center, under specific pathogen-free conditions. Mice were housed at 20–22°C, 55±5% humidity, and a 12-h light-dark cycle; water and food were provided *ad libitum*. Mice were bred and maintained in a C57BL/6 genetic background for more than 10 generations. All experimentation in mice for this project was approved by the Institutional Animal Ethical Committee (IAEC) of Biomedical Sciences Research Center “Alexander Fleming” (#376), as well as the Veterinary service and Fishery Department of the local governmental prefecture (#5365). Age and sex-matched littermate mice were assigned randomly to experimental and control groups. All measures were taken to minimize animal suffering and distress; however, neither anesthetics nor analgesics were used during the protocol, as no invasive or painful techniques were performed. The health status of the mice was monitored once per day and no unexpected deaths were observed. Mice were euthanized at predetermined time-points or when they exhibited a <20% body weight loss. Euthanasia was performed in a CO_2_ chamber with gradual filling followed by exsanguination. The generation and genotyping protocols for *Enpp2* ^n/n^ [5], R26Cre-ER^T2^ [22, 23], and R26R [24] mice have been described previously.

### Tamoxifen treatment

Tamoxifen (Tmx; Sigma T5648, USA) was dissolved in a corn oil/ ethanol (9/1) mixture at 45 mg/ml. Tmx was administered either intraperitoneally (IP; 50–100 mg/kg) or by oral gavage (Per Os, PO; 180 mg/kg), as previously described (Indra, et al., 1999, Hayashi, S. et al., 2002). Control groups received corn oil.

### Tissue processing and H&E staining

Fresh mouse tissues were fixed in 10% neutral-buffered formalin overnight at 4°C, processed using a standard overnight protocol, and embedded in paraffin blocks. Sections of 4 μm were stained with Hematoxylin and Eosin (H&E) according to standard procedures and imaged using a Nikon Eclipse E800 microscope (Nikon Corp., Shinagawa-ku, Japan) attached to a Q Imaging EXI Aqua digital camera, using the Q-Capture Pro 7 software. Images were taken and evaluated blindly.

### X-gal staining

Freshly isolated mouse tissues were embedded in OCT and frozen in liquid nitrogen. Sections of 6 μm were prepared on a cryotome and fixed in 2% formaldehyde/ 0.2% glutaraldehyde for 10 minutes at 4°C, washed twice in cold PBS /2 mM MgCl_2_ for 10 minutes and stained in X-gal staining solution (2 mg/ml X-gal (5-bromo-a-chloro-3-inodyl-β-D-galactopyranoside) in 0.1 M Na phosphate buffer pH 7.3, 0.01% Na deoxycholate, 5 mM K3Fe(CN)6, 5.7 mM K4Fe(CN)6, 2 mM MgCl_2_, 0.02% NP-40) at 37°C in the dark overnight. The sections were rinsed twice in PBS /2 mM MgCl_2_ for 10 minutes at room temperature, counterstained with eosin, and visualized under an Eclipse E800 microscope as in the previous section.

### ATX activity assay

ATX / LysoPLD activity was measured using the TOOS activity assay. ATX catalyzes the cleavage of lysophosphatidylcholine to lysophosphatidic acid and choline. The released choline is oxidised by choline oxidase to produce betaine and hydrogen peroxide. The latter is used as the oxidizing agent. In the presence of horseradish peroxidase, H_2_O_2_ reacts with TOOS (N-ethyl-N-(2-hydroxy-3-sulfopropyl)-3-methylaniline) and 4-AAP (aminoantipyrene) to form a pink quinoneimine dye which absorbs at 555 nm. 1 x LysoPLD buffer (100 mM Tris-HCl pH 9.0, 500 mM NaCl, 5 mM MgCl_2_, 5 mM CaCl_2_, 60 μM CoCl_2_, 1 mM LPC) was incubated at 37°C for 30 minutes. Plasma samples (100 fold diluted) were incubated with 1 x LysoPLD buffer at 37°C for 4 hours at a final volume of 100 μl in a 96-well plate. At the end of the incubation, a colour mix (0.5 mM 4-AAP, 7.95 U/ml HRP, 0.3 mM TOOS, 2 U/ml choline oxidase in 5 mM MgCl_2_/ 50 mM Tris-HCl pH 8.0) was prepared and 100 μl were added to each well. Absorbance (A) was measured at 555 nm every 5 minutes for 20 minutes. For each sample, the absorbance was plotted against time and the slope (dA/min) was calculated for the linear (steady-state) portion of each reaction. ATX activity was calculated according to the following equation: Activity (U/ ml) = (μmol/ min/ ml) = [dA/ min (sample)—dA/ min (blank)] * Vt/ (e* Vs* 0.5) where Vt: total volume of reaction (ml), Vs: volume of sample (ml), e: milimolar extinction coefficient of quinoneimine dye under the assay conditions (e = 32,8 μmol/ cm^2^) and 0.5: the moles of quinoneimine dye produced by 1 mol of H_2_O_2_.

### Western blotting

1 μl of plasma samples were diluted in Laemmli buffer, electrophorised on 8% SDS-polyacrylamide gels and transferred to Protran nitrocellulose membranes (Whatman plc, Maidstone, Kent, UK) using the Trans-Blot SD Semi-Dry Transfer system (Bio-Rad Laboratories, Inc., Hercules, CA, USA). Primary a-ATX Ab incubation (rat monoclonal 4F1, kindly provided by J. Aoki, 1:1000; rabbit polyclonal Cayman 1:250) was performed overnight in 5% (w/v) non-fat milk in TBS-Tween 0.05% (TBST) at 4°C. The membranes were then washed three times with TBST and incubated with HRP-conjugated secondary Abs (a-rat or a-rabbit respectively; 1:2000) for one hour at room temperature. Membranes were washed three times with TBST and antibody-antigen complexes were revealed using ECL chemiluminescent reagent (Pierce/Thermo Scientific, Rockford, IL, USA). Antibody specificity has been scrutinized previously [13].

Plasma biochemical and haematological analyses were performed with the Abbott Architect 8200 and Cell-Dyn 3700 analysers respectively.

### Real-Time RT-PCR

Total RNA was extracted from the left lung lobe using the Tri Reagent (Molecular Research Center, Inc, USA) and treated with DNAse (RQ1 RNAse-free DNAse, Promega, Wis, USA) in accordance to the manufacturer’s instructions. Reverse transcription to cDNA was performed with 3.5 μg RNA by M-MLV RT (Promega) at a final volume of 20 μl. Real-time PCR was performed on a BioRad CFX96 Touch^™^ Real-Time PCR Detection System (Bio-Rad Laboratories Ltd, CA, USA). Values were normalized to β2-microglobulin (*b2M*). The primer sequences, designated as f for forward and r for reverse, as well as the product sizes (in bp) were as follows: *Enpp2* (f, 5’-GATGCATTCCTTGTAACCAACA-3’, r, 5’- TCATCCTCAATGTCACGTAAGC-3’, 173bp), *B2M* (f, 5’-TTCTGGTGCTTGTCTCACTGA-3’, r, 5’-CAGTATGTTCGGCTTCCCATTC-3’, 104bp). The annealing temperature for all primers was 58°C.

### HPLC-MS/ MS

PF8380, LPA (C14:0, C16:0, C18:0, C18:1, C20:4), LPI (C16:0, C18:0), LPG (C16:0, C18:0), LPE (C16:0, C18:0), LPS (C16:0, C18:0) and LPC species (C14:0, C16:0, C18:0, C18:1, C24:0) were determined in plasma by HPLC-ESI/ MS/ MS using an RSLCnano system (Ultimate 3000 Series, Dionex Corporation, USA) coupled with an LTQ Orbitrap XL mass spectrometer (Thermo Scientific, Waltham, MA, USA). Plasma samples (50 μl) were mixed with 950 μl PBS and spiked with the internal standard mix (17:0 LPA/ LPC/ LPS/ LPG/ LPE). Neutral extraction was performed twice with 4 ml ice-cold CHCl_3_/ CH_3_OH (2/1, v/v) followed by 2 ml PBS saturated ice-cold CHCl_3_/ CH_3_OH (2/1, v/v). Each step of extraction was followed by a 1 minute vortex and a 1 minute centrifugation at 4°C at 3,000 rpm. The lower chloroform organic phases from both neutral extraction steps were pooled together and reserved for the LPC and PF8380 measurements. The remaining aqueous phase was chilled on ice for 10 minutes, acidified to pH 3 with acetic acid and undergone a 2 step extraction with ice-cold CHCl_3_/ CH_3_OH (2/1, v/v), as above. The lower organic phases were pooled and kept for LPA, LPS, LPG, LPI and LPE determination. The neutrally extracted organic phase and the neutralized acidified lower organic phase were evaporated to dryness. The residues were re-suspended in 0.15 ml isopropanol for HPLC-ESI/ MS/MS analysis. Obtained recoveries were 90% for PF8380, 60–85% for LPA species and 80–100% for LPC, LPS, LPG, LPI and LPE. The HPLC-MS/MS was performed as previously described [25].

### Mouse pharmacokinetics and *in vivo* inhibition of ATX activity

PF8380 was administered by oral gavage (120 mg/kg) dissolved in vehicle (Hydroxypropyl Cellulose 2% / Tween 80 0.1%) to 8 week old mice. Control mice received only the vehicle. Venus blood was collected at time points up to 12 hours post administration, in tubes containing EDTA at a final concentration of 50 mM. For plasma preparation, whole blood was centrifuged at 2000 g for 20 minutes at 4°C and the supernatant was stored in siliconized tubes at -20°C. Plasma samples were examined for ATX activity and PF8380/ LPA levels using the TOOS activity assay and HPLC- MS/MS, respectively.

### Statistical analysis

All data are expressed as means ± SEMs. Statistical analysis was performed using SigmaPlot 11.0 (Systat software Inc., IL, USA). The statistical significance was estimated in pair-wise comparisons with control values using a paired Student’s *t*-test, or a Mann-Whitney test in cases of not normal distributions. Survival curves were calculated using Kaplan-Meier survival analysis; the statistical significance of curves was assessed using Logrank test. P values <0.05 (*), p<0.01 (**) and p<0.001 (***) were considered significant. The number of samples (n) and the number of experiments/repetitions (exp) are indicated in each figure legend.

## Results and Discussion

### Inducible ubiquitous inactivation of ATX in adult mice

An inducible strategy to inactivate ATX in adult mice was used. R26Cre-ER^T2^ mice (line 2151), generated by Regeneron Pharmaceuticals [22, 23], carry a Cre recombinase-Oestrogen receptor-T2 (Cre-ER^T2^) allele targeted to the ubiquitously expressed ROSA26 (R26) locus (S1A Fig), as in previous efforts [26–28]. The ER^T2^ moiety retains the Cre recombinase in the cytoplasm until tamoxifen (Tmx; a synthetic oestrogen antagonist) administration releases this inhibition, thus permitting the nuclear translocation of Cre and the recombination of genomic loxP sites [29].

In order to confirm efficiency and tissue specificity of Tmx-induced Cre-mediated recombination, R26Cre-ER^T2^ mice were crossed with mice carrying a Cre-responsive b-galactosidase (*LacZ*) reporter allele [24]. In this reporter strain (R26R), a *lacZ* transgene has been introduced into the R26 locus, preceded by a floxed neo expression cassette preventing *lacZ* expression. Upon Cre recombinase activation, this “stop” neo cassette is removed and *lacZ* expression can be detected using X-gal staining [24]. Intraperitoneal (IP) injections of Tmx (100 mg/kg per day for 10 days) in R26Cre-ER^T2^/R26R mice resulted in varying degrees of X-gal staining in almost all tissues examined (S1B Fig), as previously reported for similar strains of mice [26–28], suggesting efficient and widespread Tmx-induced, Cre-mediated gene targeting. Moreover, no X-gal staining was detected in the absence of Tmx or the R26Cre-ER^T2^ allele (S1B Fig), indicating stringent control of gene expression.

**Fig 1:**
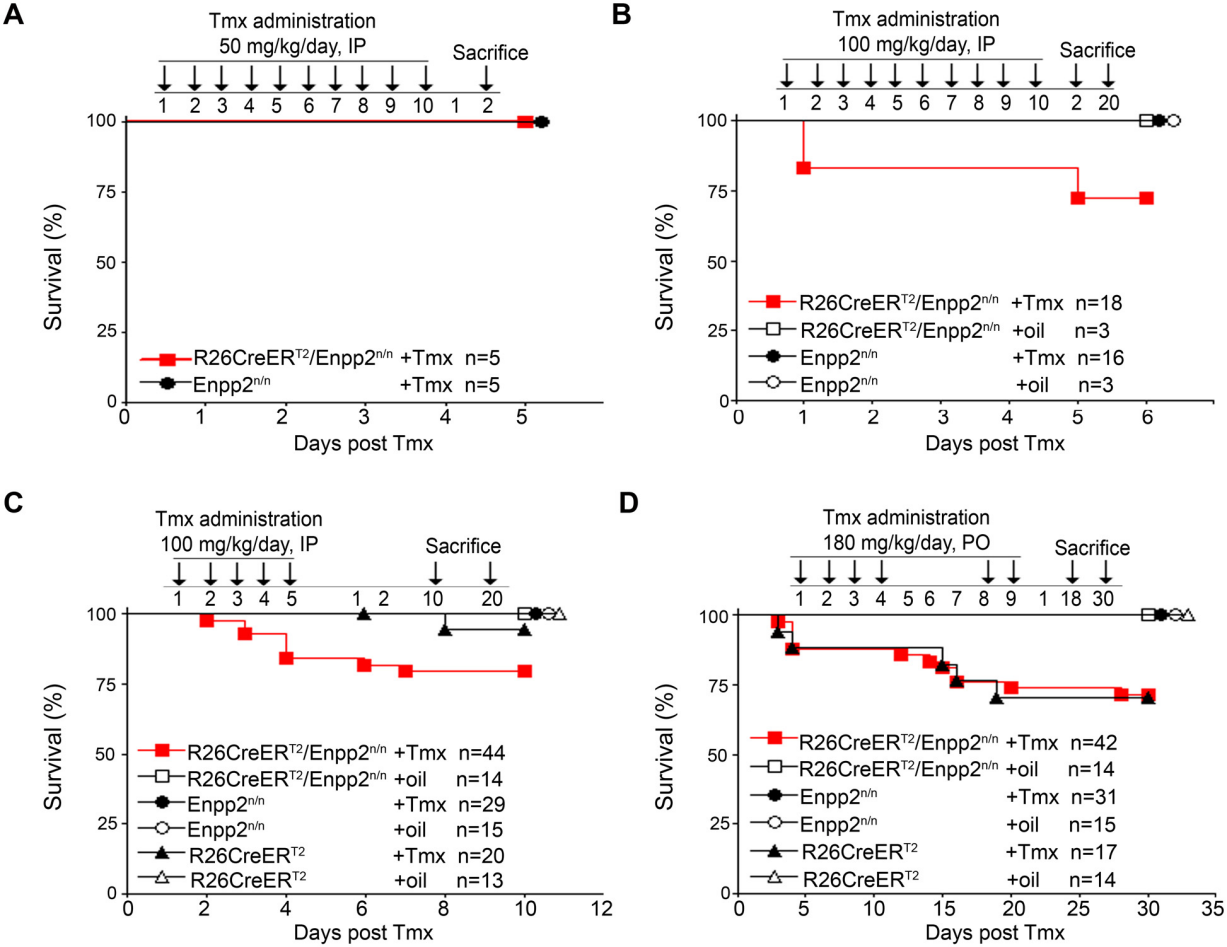
No effect of Tmx-induced genetic inactivation of ATX in mouse survival rate. Kaplan–Meier survival curves of R26Cre-ER^T2^/*Enpp2* ^n/n^ and control mice administered with different Tmx concentrations (50, 100 and 180 mg/kg) using two different routes of delivery (IP, PO), as indicated and as described in the text. Presented results are cumulative from 1 (A), 3 (B) and 4 (C and D) experiments. No statistical significant differences were observed, as assessed with the Logrank test. Overlapping curves are indicated by consecutive symbols.

For the inducible inactivation of ATX in mice *in vivo*, *Enpp2* ^n/n^ mice [5] were crossed with the R26Cre-ER^T2^ mice yielding R26Cre-ER^T2^/*Enpp2* ^n/n^ mice, which received different concentrations of Tmx via different routes of administration. Administration of 50 mg/kg Tmx IP per day for 10 days was not sufficient to induce recombination (S1 Table) and had no effect in overall mouse survival rate (Fig 1A). Increasing the Tmx dose to 100 mg/kg (IP; per day for 10 days) resulted in accurate recombination of the *Enpp2* allele, which could only be detected in the presence of the R26Cre-ER^T2^ allele (S1 Table). R26Cre-ER^T2^-mediated recombination was also detected in littermate R26Cre-ER^T2^/*Enpp2* ^n/n^ mice not receiving Tmx but housed in the same cages (see below and S1 Table) [30]. As genomic PCR is not a quantitative assay, the results from the reporter mice (S1 Fig) suggest minimal levels of leaky recombination.

No statistically significant effect in the overall survival rate of mice was observed (Fig 1B). Comparative histopathology of 44 tissues from R26Cre-ER^T2^/*Enpp2* ^n/n^ mice 2 days post Tmx administration (100 mg/kg IP per day for 10 days) at the onset of the limited observed lethality did not identify any significant findings after deletion of ATX, with the exception of lung and gastrointestinal (GI) tract which had mild to moderate inflammatory changes (S2 Table and S2 Fig) and which were followed through the entire study. However, similar histopathological changes were also observed in all control groups receiving Tmx (S2 Table and S2 Fig), while observed pathologies did not persist 20 days post Tmx administration (S3A Fig), indicating that the observed transient pathologies in the lungs and GI tract were due to Tmx administration and not ATX ablation. To lower Tmx-induced toxicity experiments were repeated using 5 days of IP injections of 100 mg/kg Tmx with similar results. *Enpp2* recombination was readily detected (S1 Table), with no statistically significant effects in mice survival rates in comparison to control mice (Fig 1C) and exhibiting no histopathological changes in lung and GI tract 10 and 20 days post Tmx treatment (S3B Fig).

### The majority of ATX activity is not necessary for adult life

To confirm results utilizing a different and more widely used route of administration, Tmx (180 mg/kg) was administered to R26Cre-ER^T2^/*Enpp2* ^n/n^ and control littermate mice with oral gavage (Per Os; PO) for 4 days, followed by a 3-day arrest and Tmx administration was continued for 2 more days (Fig 1D). Similar to the other dosing regimens reported here, Tmx-induced recombination in all tissues could be detected only in the presence of the R26Cre-ER^T2^ allele, while its minimal leakiness could be avoided with individually housing mice (S1 Table). No statistically significant effect in the overall survival rate of Tmx-administered R26Cre-ER^T2^/*Enpp2* ^n/n^ mice was observed in comparison to littermate Tmx-administered R26Cre-ER^T2^ mice, suggesting that the majority of ATX expression is dispensable for adult life and that the observed lethality could be attributed to Tmx- and Cre- mediated toxicity as previously reported [31]. No histopathological changes were detected in the lungs and GI tract of all experimental and control mice 18 and 30 days post treatment (S4 Fig). Moreover, no deleterious changes indicative of liver, kidney, or pancreatic function were identified in clinical biochemistry analytes from Tmx-administered R26Cre-ER^T2^/*Enpp2* ^n/n^ mice (S5 Fig). Likewise, no differences were detected in blood cell populations (S6 Fig). Therefore, ATX ablation appears to have no major effects in tissue homeostasis and the hematopoietic system.

**Fig 2:**
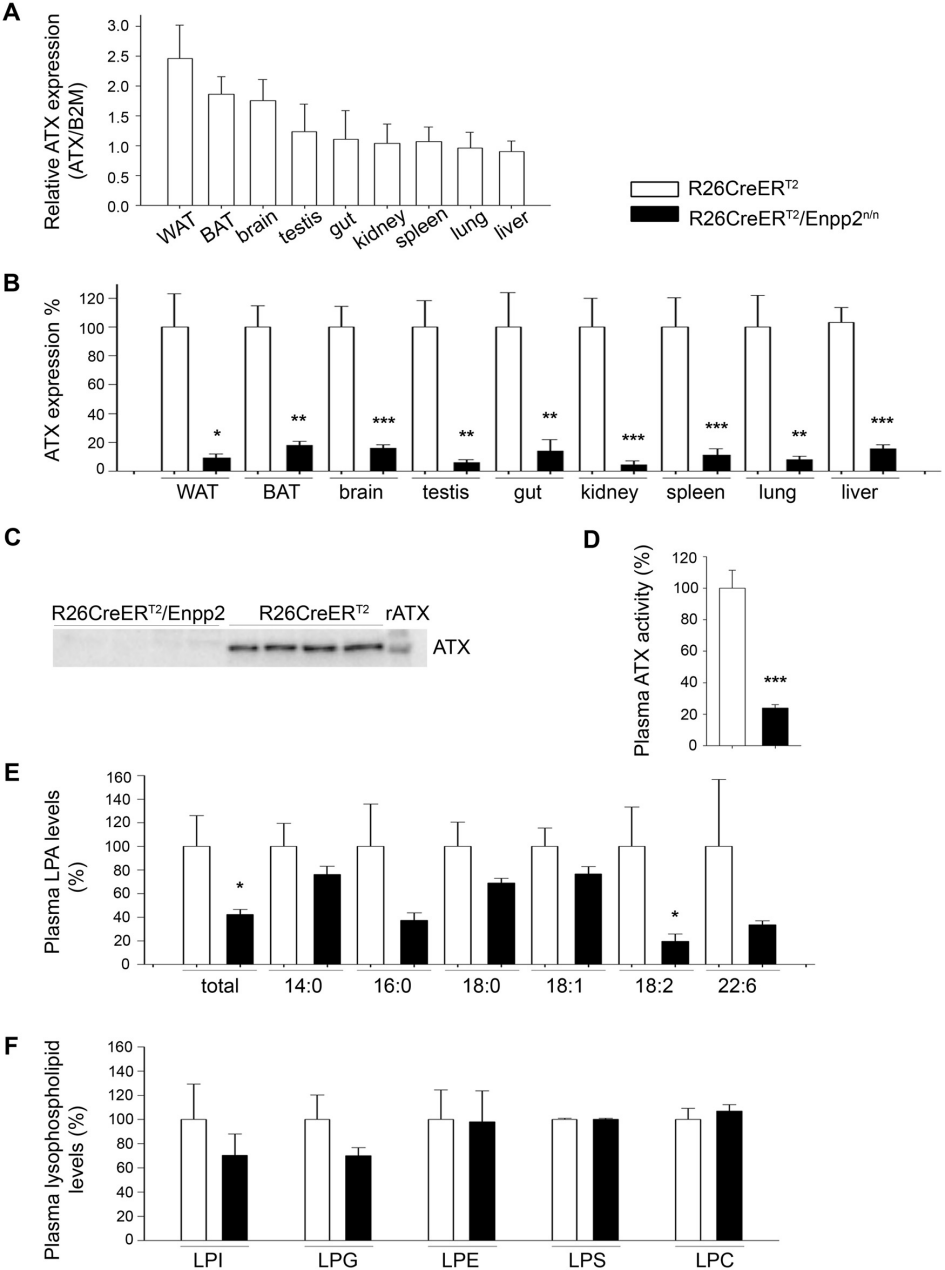
Tmx-induced (180 mg/kg PO) R26Cre-ER^T2^-mediated genetic ablation of ATX results in diminished ATX levels in tissues and plasma. (A) Real-Time RT-PCR analysis of relative ATX mRNA expression levels in different tissues normalized to the expression levels of B2M. (n = 5–10; exp = 2; with the exception of BAT/WAT n = 4, exp = 1). (B) Real-Time RT-PCR analysis of ATX mRNA expression levels, normalized to the expression levels of B2M, in different tissues following Tmx-induced genetic ablation of the *Enpp2* gene. (n = 5–10; exp = 2; with the exception of BAT/WAT n = 4, exp = 1). (C) Section from a western blot for ATX (4F1 Ab) in the plasma of the indicated mice. The full images, together with a coomassie brilliant blue staining of the same samples as loading control, and an alternate blot with a commercial antibody can be found at S7 Fig (D) Plasma ATX activity in the plasma of the indicated mice as determined with the TOOS assay on natural LPC substrates (n = 13–27; exp = 3). (E) Plasma LPA levels of the indicated mice as determined by HPLC-MS/MS (n = 9–13; exp = 2). (D) Plasma lysophospholipid (LPLs) levels remain unchanged as measured with HPLC-MS/ MS (n = 9–13; exp = 2). All values in every panel are means (± SEM) and are presented (except A) normalised (%) to control values.

Based on limited published expression studies, ATX is thought to be widely expressed in adult mice, with the highest mRNA levels reported in brain, ovary/testis, intestine, kidney, and lung [2]. Moreover, ATX has been reported to be secreted from adipocytes [32, 33], while adipose specific genetic deletion of ATX resulted in marked decreases of serum LPA [34], suggesting the adipose tissue as a major contributor to serum ATX. Real Time RT-PCR analysis of ATX mRNA expression confirmed the brain and adipose (gonadal white adipose tissue, WAT; brown adipose tissue, BAT) tissues as the highest ATX expression tissues in wt C57BL/6 mice (Fig 2A), in general agreement with on line expression data repositories (http://biogps.org/#goto=genereport&id=18606, http://www.gtexportal.org/home/gene/ENPP2). Tmx-induced, R26Cre-ER^T2^-mediated *Enpp2* recombination resulted in >80% attenuation of ATX mRNA levels in all organs examined including brain and adipose tissue (Fig 2B), as quantified with Real-Time RT-PCR. As a consequence, ATX protein was barely detectable in the plasma of Tmx-administered R26Cre-ER^T2^/*Enpp2* ^n/n^ mice (Fig 2C and S7 Fig), resulting to an 80% decrease of plasma ATX activity levels (Fig 2D), as quantified with the TOOS assay on natural LPC substrates. ATX activity levels were followed by a similar decrease in LPA levels (Fig 2E) despite the unaffected levels of possible ATX substrates (Fig 2F), as quantified by HPLC-MS/MS.

A 50% reduction in plasma ATX (and LPA) levels in heterozygous ATX KO mice was previously reported to result in no overt phenotype [5–7]. Our results extend these observations proving that even an 80% reduction in ATX activity and LPA levels in adult mice has no effect on tissue histopathology and overall survival. However, we cannot exclude the possibility that residual total LPA may still be enough to maintain normal body functions, although the reported LPA concentration thresholds to observe any effect in any cell type *in vitro* is much higher (>>1 μM up to 10 μM)[2]. In fact, most reported effects of LPA *in vitro* were observed at non-physiological concentrations corresponding to pathophysiological situations of increased local concentrations of LPA at inflamed/malignant sites, most likely as a result of increased expression of ATX. Moreover, it should be noted that cellular effects of LPA will depend not only on its local concentration (controlled by the relative levels of both ATX and LPPs), but as well as on the local cell-specific abundance of LPA receptors and their suggested transactivation by other factors [35] or the local concentration of interacting proteins that have been reported to modulate its biological effects [36]. Additionally and depending on the concentration, LPA may also elicit cellular responses not only through specific binding to cognate receptors, but as well as through the activation of nuclear receptors [37], through the modulation of macromolecular signalling complexes via direct docking to proteins or the metabolic rewiring of cell metabolism [38].

### Potent pharmacological ATX inhibition exhibits no toxic effects

Upregulated expression of ATX has been detected in a number of chronic inflammatory diseases and cancer, while genetic interventions have proven a role of ATX in disease pathogenesis; establishing ATX as a promising potential therapeutic target [2]. However, the embryonic lethality of ATX knockout mice poses a concern for the suitability of ATX as a drug target.

**Fig 3:**
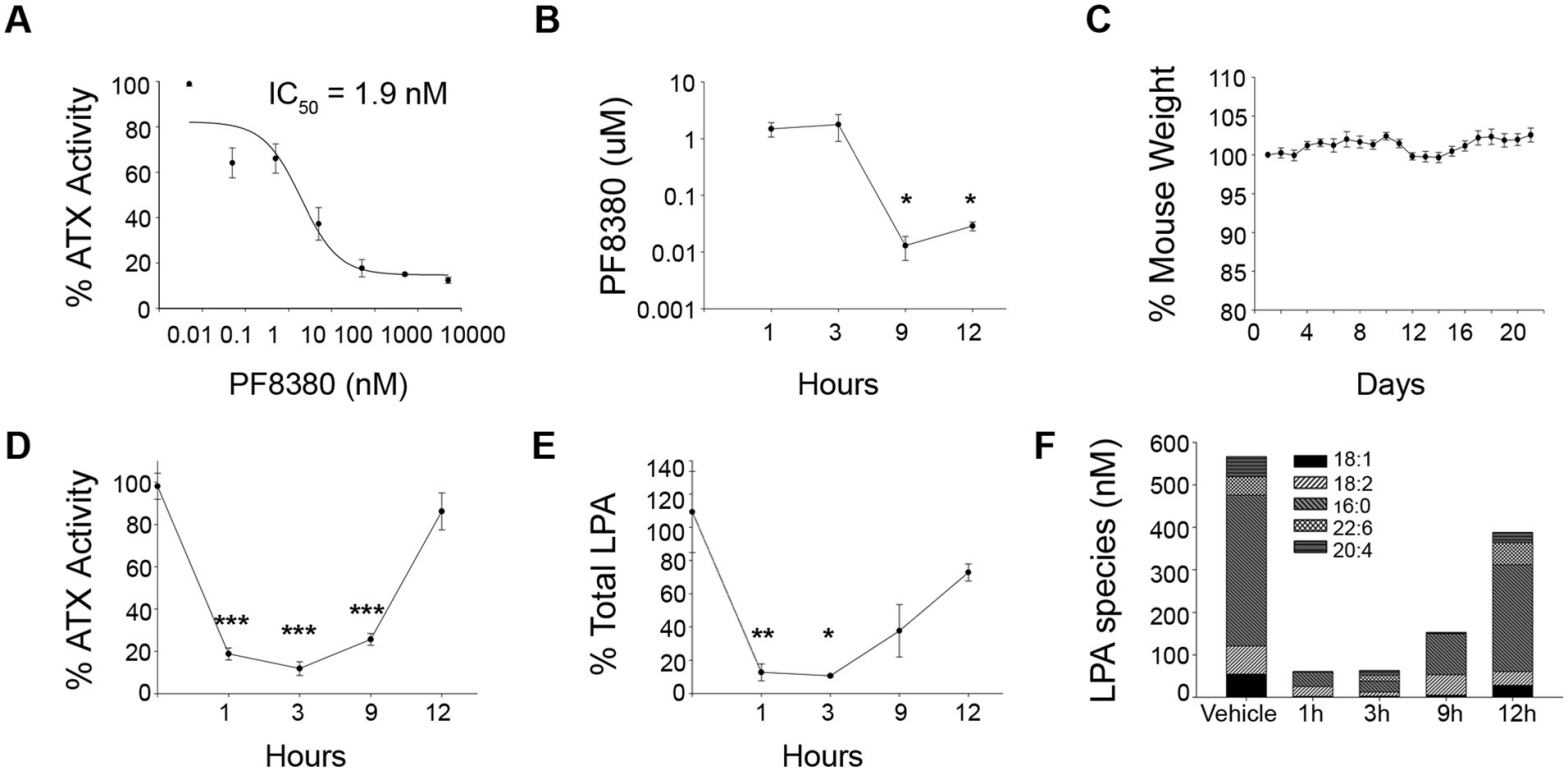
PF8380 inhibits ATX activity *in vitro* and *in vivo*. (A) Dose response ATX activity inhibition curve under various PF8380 concentrations as measured with the TOOS assay using 50 μM of 16:0 LPC as substrate. (B) Plasma PF8380 pharmacokinetic profile following administration of 120 mg/kg PF8380 for 3 weeks, by oral gavage, in female mice. (C) No effect in mouse body weight following treatment with PF8380 as above. (D) Percent (%) residual plasma ATX activity measured in the presence of 1 mM 16:0 LPC with the TOOS assay at various time points post PF8380 administration as above. (E) Percent (%) residual plasma total LPA levels and (F) concentration of different LPA species at different time points post treatment with PF8380 as above. The cumulative values of two independent experiments (n = 4–7) are presented as means (± SEMs).

Inducible, ubiquitous genetic deletion of ATX indicated that the majority of ATX expression is dispensable for adult life, suggesting potential safety of pharmaceutical targeting. To validate the conclusion, mice were treated with the potent ATX inhibitor 6-(3-(piperazin-1-yl)propanoyl)-benzo[d]oxazol-2(3H)-one (PF8380)[39]. PF8380 is small molecule with a long linear and flexible structure, good oral bioavailability, and a reported IC50 of 1.7 nM on natural LPC substrates [39]. Oral administration of 30 mg/kg PF8380 reduced inflammatory hyperalgesia in a rat air pouch model, exhibiting >95% reduction of LPA levels in both plasma and inflammatory site tissue within 3 hours [39]. *In vitro* testing on natural LPC substrates of synthesized PF8380 confirmed its potency (IC50 1.9 nM) (Fig 3A), while pharmacokinetic profiling indicated that its levels remained above IC50 values for at least 12 hours (Fig 3B). Wild type 8 week old mice were treated twice daily for 3 weeks with 120 mg/kg PF8380 administered by oral gavage (PO). Despite high dosing for a prolonged period, treated mice exhibited no weight loss (Fig 3C) or macroscopic signs of toxicity. Histopathological examination of 13 major organs following treatment (S8 Fig) identified no pathological changes, confirming that the bulk of ATX activity is dispensable for adult life and that ATX is a safe therapeutic target. Similar conclusions have been reached following administration of ONO-8430506, a novel ATX inhibitor (reported IC50 6.4–19 nM)[40], for 4 or 21 days at a concentration 10 or 100 mg/kg, where no lethality or weight loss was observed [41].

## Conclusions

Given the extensive steady state reduction of LPA levels upon inducible genetic deletion of ATX and the pharmacokinetics (PK) and pharmacodynamics (PD) profiles of existing potent ATX inhibitors, it is highly unlikely to achieve experimentally a further, 24 hours-long, reduction in LPA levels by combining genetic and pharmacologic approaches. However, such a hypothetical 100% constant ATX inhibition would not only prove that the entire ATX expression and activity (as opposed to 80% shown here) is dispensable for adult life, but would also indicate the relative contribution to serum LPA levels from alternative synthetic pathways possibly mediated by phospholipase A-type enzymes as previously suggested [42].

As the important role of ATX in chronic inflammation and cancer is emerging, a large number of ATX inhibitors have been developed over the last years [2, 20], while several of them have exhibited therapeutic potential: in air-pouch inflammation [39], collagen-induced arthritis [43], bleomycin-induced pulmonary inflammation and fibrosis [14], allergen-induced asthma [44], as well as in the metastasis of melanoma and breast cancer cells [41, 45–48]. Moreover, ATX inhibition has been proposed as a potential adjuvant therapy to known cancer treatments, given its suggested role in chemotherapy and radiotherapy resistance [49, 50]. Noteworthy, the therapeutic effects of inhibiting ATX in most reported pharmacological studies did not always correlate with the PK/PD profile of the compound, when available, and LPA levels were never suppressed >80% for more than three hours. Although the tissue penetration and retention of the compounds and tissue LPA levels post treatment are largely unknown, it’s safe to conclude that even the most potent ATX inhibitors that do exhibit therapeutic potential cannot attenuate LPA levels >80% at a steady state for 24 hours. On the other hand the present study has shown that even a constant 80% reduction of LPA is well tolerated systemically. Therefore, ATX inhibition can exert its therapeutic benefits by inhibiting, most likely, the increased amounts of ATX at inflamed and malignant sites locally, without exhibiting systemic, toxic side effects.

## Supporting Information

S1 Fig Widespread sporadic Cre excision in tamoxifen-treated R26CreER^T2^/ROSAlacZ mice. **A**. Schematic representation of the gene targeting strategy. The R26Cre-ER^T2^ construct was sequentially inserted into the *Rosa26* locus by homologous recombination as depicted. E, EcoRI; P, PacI; CreER^T2^, adenovirus splice acceptor-CreER^T2^-PMC polyadenylation site; H, Hygro, PGK-hygromycin. **B**. Tissue sections from 8-week old double transgenic R26Cre-ER^T2^/R26R mice and littermates were stained for b-galactosidase activity to identify sites of active Cre-mediated recombination. (Scale bar: 150 μm).
(PDF)

S2 Fig *In vivo* Tmx treatment results in minor inflammatory changes in lung and gastrointestinal (GI) tract. Representative images of tissue sections (H&E staining) from R26Cre-ER^T2^/*Enpp2* ^n/n^ mice and littermates treated IP with Tmx (100 mg/kg) or corn oil once per day for 10 days. Mice were sacrificed 2 days post Tmx treatment. (Scale bar: 50 μm). Mice treated with corn oil had no histopathological changes indicating that the inflammatory changes were due to Tmx administration.
(PDF)

S3 Fig Genetic ablation of *Enpp2* has no effect in tissue histology. Representative images of tissue sections (H&E staining) from R26Cre-ER^T2^/*Enpp2* ^n/n^ mice and littermates treated IP with Tmx (100 mg/kg) or corn oil for **A**. 10 days and **B**. 5 days. Mice were sacrificed 20 or 10 and 20 days post Tmx treatment, respectively. (Scale bar: 150 μm).
(PDF)

S4 Fig Genetic deletion of *Enpp2* has no effect in tissue histology. Representative images of tissue sections (H&E staining) from R26Cre-ER^T2^/*Enpp2* ^n/n^ mice and littermates treated PO with Tmx (180 mg/kg) or corn oil for 6 days. Mice were sacrificed 18 and 30 days post Tmx administration. (Scale bar: 150 μm).
(PDF)

S5 Fig Genetic excision of *Enpp2* has no effect in biochemical factors indicative of main body functions. Plasma biochemical analytes from Tmx / oil-administered R26Cre-ER^T2^/*Enpp2* ^n/n^ mice and littermates (n = 3–8, exp = 2). AST: Aspartate transaminase; ALT: Alanine transaminase; CPK: Creatine phosphokinase; LDH: Lactate dehydrogenase; γ-GT: γ-glutamyl-transpeptidase.
(PDF)

S6 Fig Genetic excision of *Enpp2* has no effect in hematopoietic cell populations. Blood cell counts and parameters in Tmx-treated R26Cre-ER^T2^/*Enpp2* ^n/n^ mice and littermates (n = 3–6, exp = 1). WBC: White blood cells; LYMPH: Lymphocytes; MXD: monocytes, basophils and eosinophils; GRA: Granulocytes; RBC: Red blood cells; HGB: hemoglobin; HCT: hematocrit; MCV: mean volume of erythrocytes; MCH: mean content of hemoglobin; MCHC: mean concentration of hemoglobin; RDW: Red cell Distribution Width; PLT: platelets; PCT: plateletkrit; MPV: mean platelet volume; PDW: relative width of the distribution of platelets.
(PDF)

S7 Fig Genetic excision of *Enpp2* attenuates its protein levels in the plasma. (A) Western blot of the indicated mouse plasma samples with a rat monoclonal antibody (4F1) against ATX. (B) Western blot of the same (as in A) samples with a commercial (Cayman) rabbit polyclonal against ATX. (C) Coomassie brilliant blue staining of the same (as in A) samples, serving as a loading control.
(PDF)

S8 Fig Potent pharmacological inhibition of ATX has no effect on tissue histology. Representative images of tissue sections from vehicle-treated and PF8380-treated mice (120 mg/kg PF8380, PO, twice a day for 3 weeks), stained with H&E. (Scale bar: 150 μm).
(PDF)

S1 Table R26CreER^T2^-driven *Enpp2* recombination, as tested by PCR, in the indicated mouse strains and upon the indicated treatments. (PDF)

S2 Table No major effects in tissue physiology upon inducible, complete genetic deletion of ATX. (PDF)

## References

[1] Umezu-Goto M, Kishi Y, Taira A, Hama K, Dohmae N, Takio K, et al Autotaxin has lysophospholipase D activity leading to tumor cell growth and motility by lysophosphatidic acid production. The Journal of cell biology. 2002;158(2):227–33. 10.1083/jcb.200204026 12119361 PMC2173129

[2] Barbayianni E, Kaffe E, Aidinis V, Kokotos G. Autotaxin, a secreted lysophospholipase D, as a promising therapeutic target in chronic inflammation and cancer. Progress in lipid research. 2015;58:76–96. Epub 2015/02/24. 10.1016/j.plipres.2015.02.001 . 25704398

[3] Perrakis A, Moolenaar WH. Autotaxin: structure-function and signaling. J Lipid Res. 2014 Epub 2014/02/20. 10.1194/jlr.R046391 . 24548887 PMC4031933

[4] Nakanaga K, Hama K, Aoki J. Autotaxin—an LPA producing enzyme with diverse functions. J Biochem. 2010;148(1):13–24. Epub 2010/05/25. 10.1093/jb/mvq052 . 20495010

[5] Fotopoulou S, Oikonomou N, Grigorieva E, Nikitopoulou I, Paparountas T, Thanassopoulou A, et al ATX expression and LPA signalling are vital for the development of the nervous system. Dev Biol. 2010;339(2):451–64. 10.1016/j.ydbio.2010.01.007 20079728

[6] Tanaka M, Okudaira S, Kishi Y, Ohkawa R, Iseki S, Ota M, et al Autotaxin stabilizes blood vessels and is required for embryonic vasculature by producing lysophosphatidic acid. J Biol Chem. 2006;281(35):25822–30. . 16829511 10.1074/jbc.M605142200

[7] van Meeteren LA, Ruurs P, Stortelers C, Bouwman P, van Rooijen MA, Pradere JP, et al Autotaxin, a secreted lysophospholipase D, is essential for blood vessel formation during development. Molecular and cellular biology. 2006;26(13):5015–22. . 16782887 10.1128/MCB.02419-05 PMC1489177

[8] Okudaira S, Yukiura H, Aoki J. Biological roles of lysophosphatidic acid signaling through its production by autotaxin. Biochimie. 2010;92(6):698–706. Epub 2010/04/27. S0300-9084(10)00153-7 [pii] 10.1016/j.biochi.2010.04.015 . 20417246

[9] Choi JW, Herr DR, Noguchi K, Yung YC, Lee CW, Mutoh T, et al LPA receptors: subtypes and biological actions. Annu Rev Pharmacol Toxicol. 2010;50:157–86. Epub 2010/01/09. 10.1146/annurev.pharmtox.010909.105753 . 20055701

[10] Yanagida K, Kurikawa Y, Shimizu T, Ishii S. Current progress in non-Edg family LPA receptor research. Biochim Biophys Acta. 2013;1831(1):33–41. Epub 2012/08/21. S1388-1981(12)00168-0 [pii] 10.1016/j.bbalip.2012.08.003 . 22902318

[11] Sevastou I, Kaffe E, Mouratis MA, Aidinis V. Lysoglycerophospholipids in chronic inflammatory disorders: The PLA(2)/LPC and ATX/LPA axes. Biochim Biophys Acta. 2013;1831(1):42–60. Epub 2012/08/08. S1388-1981(12)00154-0 [pii] 10.1016/j.bbalip.2012.07.019 . 22867755

[12] Benesch MG, Ko YM, McMullen TP, Brindley DN. Autotaxin in the crosshairs: Taking aim at cancer and other inflammatory conditions. FEBS letters. 2014 Epub 2014/02/25. 10.1016/j.febslet.2014.02.009 . 24560789

[13] Nikitopoulou I, Oikonomou N, Karouzakis E, Sevastou I, Nikolaidou-Katsaridou N, Zhao Z, et al Autotaxin expression from synovial fibroblasts is essential for the pathogenesis of modeled arthritis. J Exp Med. 2012;209(5):925–33. Epub 2012/04/12. jem.20112012 [pii] 10.1084/jem.20112012 . 22493518 PMC3348105

[14] Oikonomou N, Mouratis MA, Tzouvelekis A, Kaffe E, Valavanis C, Vilaras G, et al Pulmonary autotaxin expression contributes to the pathogenesis of pulmonary fibrosis. American journal of respiratory cell and molecular biology. 2012;47(5):566–74. Epub 2012/06/30. 10.1165/rcmb.2012-0004OC . 22744859

[15] Bourgoin SG, Zhao C. Autotaxin and lysophospholipids in rheumatoid arthritis. Curr Opin Investig Drugs. 2010;11(5):515–26. Epub 2010/04/27. . 20419597

[16] Tager AM. Autotaxin emerges as a therapeutic target for idiopathic pulmonary fibrosis: limiting fibrosis by limiting lysophosphatidic acid synthesis. American journal of respiratory cell and molecular biology. 2012;47(5):563–5. Epub 2012/11/06. 10.1165/rcmb.2012-0235ED 23125419 PMC3547105

[17] Liu S, Umezu-Goto M, Murph M, Lu Y, Liu W, Zhang F, et al Expression of autotaxin and lysophosphatidic acid receptors increases mammary tumorigenesis, invasion, and metastases. Cancer Cell. 2009;15(6):539–50. 10.1016/j.ccr.2009.03.027 19477432 PMC4157573

[18] Hausmann J, Kamtekar S, Christodoulou E, Day JE, Wu T, Fulkerson Z, et al Structural basis of substrate discrimination and integrin binding by autotaxin. Nat Struct Mol Biol. 2011;18(2):198–204. 10.1038/nsmb.1980 21240271 PMC3064516

[19] Albers HM, Ovaa H. Chemical Evolution of Autotaxin Inhibitors. Chem Rev. 2012 Epub 2012/02/18. 10.1021/cr2003213 . 22335786 PMC3349290

[20] Barbayianni E, Magrioti V, Moutevelis-Minakakis P, Kokotos G. Autotaxin inhibitors: a patent review. Expert Opin Ther Pat. 2013 Epub 2013/05/07. 10.1517/13543776.2013.796364 . 23641951

[21] Nishimasu H, Okudaira S, Hama K, Mihara E, Dohmae N, Inoue A, et al Crystal structure of autotaxin and insight into GPCR activation by lipid mediators. Nat Struct Mol Biol. 2011;18(2):205–12. Epub 2011/01/18. nsmb.1998 [pii] 10.1038/nsmb.1998 . 21240269

[22] Economides AN, Frendewey D, Yang P, Dominguez MG, Dore AT, Lobov IB, et al Conditionals by inversion provide a universal method for the generation of conditional alleles. Proc Natl Acad Sci U S A. 2013;110(34):E3179–88. Epub 2013/08/07. 10.1073/pnas.1217812110 23918385 PMC3752204

[23] Higashi AY, Ikawa T, Muramatsu M, Economides AN, Niwa A, Okuda T, et al Direct hematological toxicity and illegitimate chromosomal recombination caused by the systemic activation of CreERT2. J Immunol. 2009;182(9):5633–40. Epub 2009/04/22. 10.4049/jimmunol.0802413 . 19380810

[24] Soriano P. Generalized lacZ expression with the ROSA26 Cre reporter strain. Nat Genet. 1999;21(1):70–1. Epub 1999/01/23. 10.1038/5007 . 9916792

[25] Nikitopoulou I, Kaffe E, Sevastou I, Sirioti I, Samiotaki M, Madan D, et al A metabolically-stabilized phosphonate analog of lysophosphatidic acid attenuates collagen-induced arthritis. PLoS One. 2013;8(7):e70941 10.1371/journal.pone.0070941. Print 2013. 23923032 PMC3726599

[26] Hameyer D, Loonstra A, Eshkind L, Schmitt S, Antunes C, Groen A, et al Toxicity of ligand-dependent Cre recombinases and generation of a conditional Cre deleter mouse allowing mosaic recombination in peripheral tissues. Physiol Genomics. 2007;31(1):32–41. Epub 2007/04/26. 10.1152/physiolgenomics.00019.2007 . 17456738

[27] Seibler J, Zevnik B, Kuter-Luks B, Andreas S, Kern H, Hennek T, et al Rapid generation of inducible mouse mutants. Nucleic Acids Res. 2003;31(4):e12 Epub 2003/02/13. 12582257 10.1093/nar/gng012 PMC150244

[28] Ventura A, Kirsch DG, McLaughlin ME, Tuveson DA, Grimm J, Lintault L, et al Restoration of p53 function leads to tumour regression in vivo. Nature. 2007;445(7128):661–5. Epub 2007/01/26. 10.1038/nature05541 . 17251932

[29] Feil R, Wagner J, Metzger D, Chambon P. Regulation of Cre recombinase activity by mutated estrogen receptor ligand-binding domains. Biochem Biophys Res Commun. 1997;237(3):752–7. Epub 1997/08/28. 10.1006/bbrc.1997.7124 . 9299439

[30] Brake RL, Simmons PJ, Begley CG. Cross-contamination with tamoxifen induces transgene expression in non-exposed inducible transgenic mice. Genet Mol Res. 2004;3(4):456–62. Epub 2005/02/03. . 15688312

[31] Schmidt-Supprian M, Rajewsky K. Vagaries of conditional gene targeting. Nat Immunol. 2007;8(7):665–8. Epub 2007/06/21. 10.1038/ni0707-665 . 17579640

[32] Boucharaba A, Serre CM, Gres S, Saulnier-Blache JS, Bordet JC, Guglielmi J, et al Platelet-derived lysophosphatidic acid supports the progression of osteolytic bone metastases in breast cancer. The Journal of clinical investigation. 2004;114(12):1714–25. . 15599396 10.1172/JCI22123 PMC535068

[33] Saulnier-Blache JS. [Lysophosphatidic acid: a "bioactive" phospholipid]. Med Sci (Paris). 2004;20(8–9):799–803. . 15361348 10.1051/medsci/2004208-9799

[34] Dusaulcy R, Rancoule C, Gres S, Wanecq E, Colom A, Guigne C, et al Adipose-specific disruption of autotaxin enhances nutritional fattening and reduces plasma lysophosphatidic acid. J Lipid Res. 2011;52(6):1247–55. Epub 2011/03/23. jlr.M014985 [pii] 10.1194/jlr.M014985 21421848 PMC3090245

[35] Rai V, Toure F, Chitayat S, Pei R, Song F, Li Q, et al Lysophosphatidic acid targets vascular and oncogenic pathways via RAGE signaling. J Exp Med. 2012;209(13):2339–50. Epub 2012/12/05. 10.1084/jem.20120873 23209312 PMC3526353

[36] Tigyi G, Parrill AL. Molecular mechanisms of lysophosphatidic acid action. Progress in lipid research. 2003;42(6):498–526. Epub 2003/10/16. . 14559069 10.1016/s0163-7827(03)00035-3

[37] McIntyre TM, Pontsler AV, Silva AR, St Hilaire A, Xu Y, Hinshaw JC, et al Identification of an intracellular receptor for lysophosphatidic acid (LPA): LPA is a transcellular PPARgamma agonist. Proc Natl Acad Sci U S A. 2003;100(1):131–6. Epub 2002/12/28. [pii]. 12502787 10.1073/pnas.0135855100 PMC140905

[38] Parrill AL, Tigyi G. Integrating the puzzle pieces: The current atomistic picture of phospholipid-G protein coupled receptor interactions. Biochim Biophys Acta. 2013;1831(1):2–12. Epub 2012/09/18. S1388-1981(12)00199-0 [pii] 10.1016/j.bbalip.2012.09.002 . 22982815 PMC3591812

[39] Gierse JK, Thorarensen A, Beltey K, Bradshaw-Pierce E, Cortes-Burgos L, Hall T, et al A Novel Autotaxin Inhibitor Reduces Lysophosphatidic Acid Levels in Plasma and the Site of Inflammation. The Journal of pharmacology and experimental therapeutics. 2010 . 20392816 10.1124/jpet.110.165845

[40] Saga H, Ohhata A, Hayashi A, Katoh M, Maeda T, Mizuno H, et al A Novel Highly Potent Autotaxin/ENPP2 Inhibitor Produces Prolonged Decreases in Plasma Lysophosphatidic Acid Formation In Vivo and Regulates Urethral Tension. PLoS One. 2014;9(4):e93230 Epub 2014/04/22. 10.1371/journal.pone.0093230 24747415 PMC3991570

[41] Benesch MG, Tang X, Maeda T, Ohhata A, Zhao YY, Kok BP, et al Inhibition of autotaxin delays breast tumor growth and lung metastasis in mice. Faseb J. 2014 Epub 2014/03/07. 10.1096/fj.13-248641 . 24599971

[42] Aoki J, Inoue A, Okudaira S. Two pathways for lysophosphatidic acid production. Biochim Biophys Acta. 2008;1781(9):513–8. 10.1016/j.bbalip.2008.06.005 18621144

[43] Nikitopoulou I, Sevastou I, Madan D, Prestwich GD, Aidinis V. A bromo-phosphonate analogue of lysophosphatidic acid attenuates the development of collagen induced arthritis. PLoS One. 2013;in press. 10.1371/journal.pone.0070941 PMC3726599 23923032

[44] Park GY, Lee YG, Berdyshev E, Nyenhuis S, Du J, Fu P, et al Autotaxin production of Lysophosphatidic Acid Mediates Allergic Asthmatic Inflammation. Am J Respir Crit Care Med. 2013 Epub 2013/09/21. 10.1164/rccm.201306-1014OC . 24050723 PMC3826286

[45] Zhang H, Xu X, Gajewiak J, Tsukahara R, Fujiwara Y, Liu J, et al Dual activity lysophosphatidic acid receptor pan-antagonist/autotaxin inhibitor reduces breast cancer cell migration in vitro and causes tumor regression in vivo. Cancer research. 2009;69(13):5441–9. 10.1158/0008-5472.CAN-09-0302 19509223 PMC3446773

[46] Lee SC, Fujiwara Y, Liu J, Yue J, Shimizu Y, Norman DD, et al Autotaxin, LPA Receptors (1 and 5) Exert Disparate Functions in Tumor Cells Versus the Host Tissue Microenvironment in Melanoma Invasion and Metastasis. Mol Cancer Res. 2014 Epub 2014/08/28. . 25158955 10.1158/1541-7786.MCR-14-0263 PMC4297753

[47] Leblanc R, Lee SC, David M, Bordet JC, Norman DD, Patil R, et al Interaction of platelet-derived autotaxin with tumor integrin alphaVbeta3 controls metastasis of breast cancer cells to bone. Blood. 2014 Epub 2014/10/04. 10.1182/blood-2014-04-568683 . 25277122 PMC4231421

[48] Benesch MG, Tang X, Dewald J, Dong WF, Mackey JR, Hemmings DG, et al Tumor-induced inflammation in mammary adipose tissue stimulates a vicious cycle of autotaxin expression and breast cancer progression. Faseb J. 2015 Epub 2015/06/14. 10.1096/fj.15-274480 . 26071407

[49] Brindley DN, Lin FT, Tigyi GJ. Role of the autotaxin-lysophosphatidate axis in cancer resistance to chemotherapy and radiotherapy. Biochim Biophys Acta. 2012 Epub 2012/09/08. S1388-1981(12)00186-2 [pii] 10.1016/j.bbalip.2012.08.015 . 22954454 PMC3584168

[50] Venkatraman G, Benesch MG, Tang X, Dewald J, McMullen TP, Brindley DN. Lysophosphatidate signaling stabilizes Nrf2 and increases the expression of genes involved in drug resistance and oxidative stress responses: implications for cancer treatment. Faseb J. 2014 Epub 2014/11/16. 10.1096/fj.14-262659 . 25398768

