# Supplementary material for: The Bulk of Autotaxin Activity Is Dispensable for Adult Mouse Life
Source: PLoS One. 2015 Nov 16;10(11):e0143083. doi: 10.1371/journal.pone.0143083 (PMC4646642; doi:10.1371/journal.pone.0143083)
Supplement: S1 Fig — A. Schematic representation of the gene targeting strategy. The R26Cre-ERT2 construct was sequentially inserted into the Rosa26 locus by homologous recombination as depicted. E, EcoRI; P, PacI; CreERT2, adenovirus splice acceptor-CreERT2-PMC polyadenylation site; H, Hygro, PGK-hygromycin. B. Tissue sections from 8-week old double transgenic R26Cre-ERT2/R26R mice and littermates were stained for b-galactosidase activity to identify sites of active Cre-mediated recombination. (Scale bar: 150 μm). (PDF) [file pone.0143083.s001.pdf]

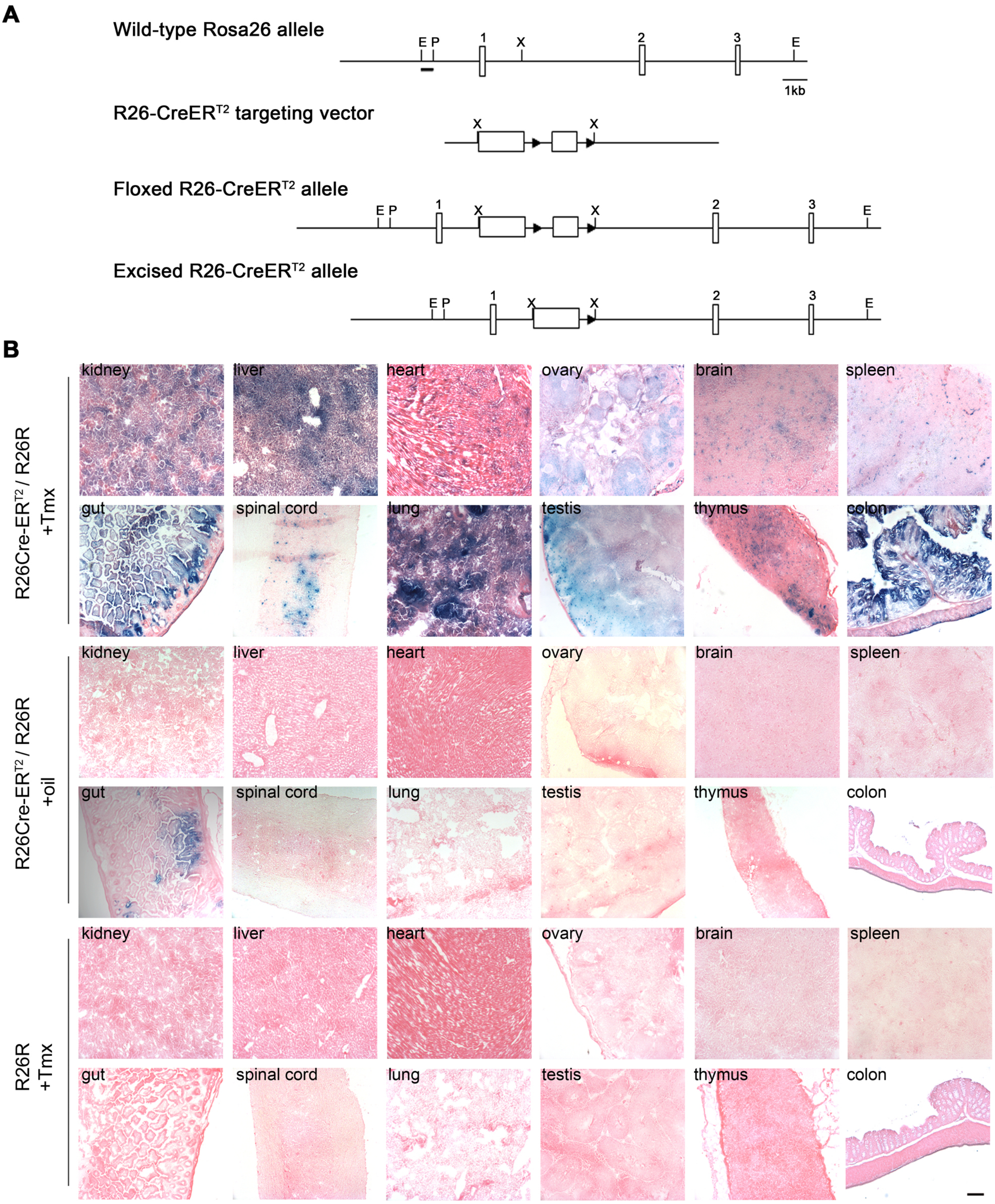

S1 Fig. Widespread sporadic Cre excision in tamoxifen-treated R26CreER^{T2}/ROSAlacZ mice. A. Schematic representation of the gene targeting strategy. The R26Cre-ER^{T2} construct was sequentially inserted into the Rosa26 locus by homologous recombination as depicted. E, EcoRI; P, PacI; CreER^{T2}, adenovirus splice acceptor-CreER^{T2}-PMC polyadenylation site; H, Hygro, PGK-hygromycin. **B.** Tissue sections from 8-week old double transgenic R26Cre-ER^{T2}/R26R mice and littermates were stained for b-galactosidase activity to identify sites of active Cre- mediated recombination (Scale bar: 150 μm).
