# Supplementary material for: The Bulk of Autotaxin Activity Is Dispensable for Adult Mouse Life
Source: PLoS One. 2015 Nov 16;10(11):e0143083. doi: 10.1371/journal.pone.0143083 (PMC4646642; doi:10.1371/journal.pone.0143083)
Supplement: S2 Fig — Representative images of tissue sections (H&E staining) from R26Cre-ERT2/Enpp2 n/n mice and littermates treated IP with Tmx (100 mg/kg) or corn oil once per day for 10 days. Mice were sacrificed 2 days post Tmx treatment. (Scale bar: 50 μm). Mice treated with corn oil had no histopathological changes indicating that the inflammatory changes were due to Tmx administration. (PDF) [file pone.0143083.s002.pdf]

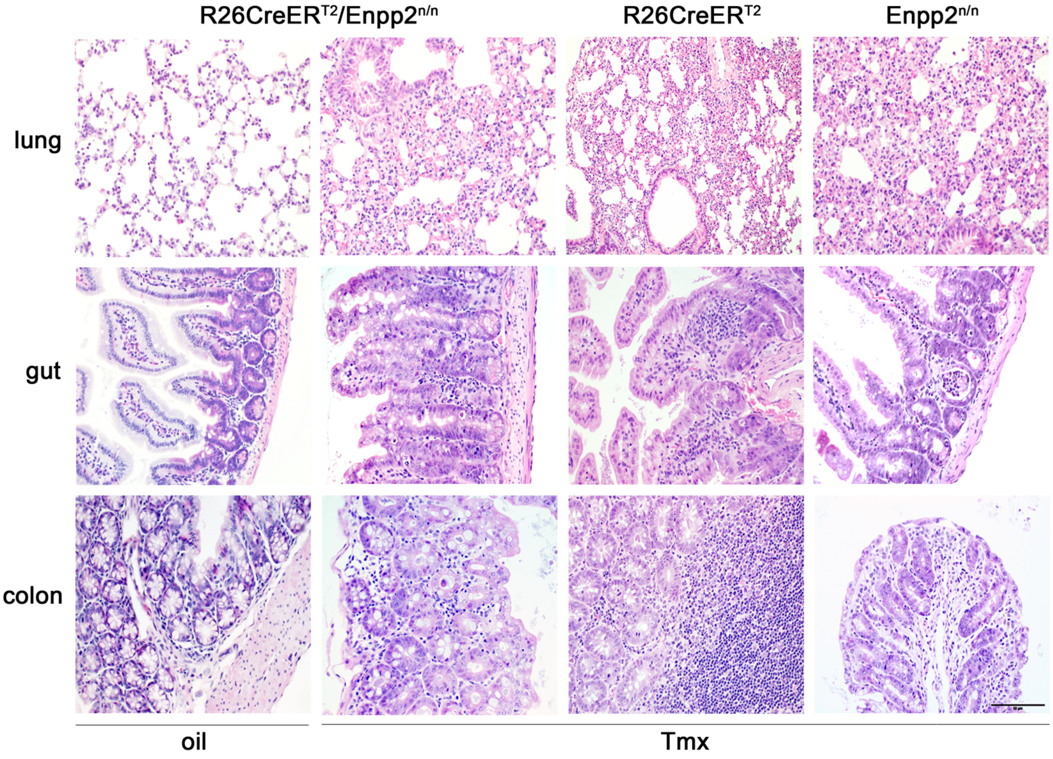

S2 Fig. *In vivo* Tmx treatment results in minor inflammatory changes in lung and gastrointestinal (GI) tract. Representative images of tissue sections (H&E staining) from R26Cre-ER^{T2}/Enpp2$^{n/n}$ mice and littermates treated IP with Tmx (100 mg/kg) or corn oil once per day for 10 days. Mice were sacrificed 2 days post Tmx treatment. (Scale bar: 50 μm). Mice treated with corn oil had no histopathological changes indicating that the inflammatory changes were due to Tmx administration.
