# Supplementary material for: The Bulk of Autotaxin Activity Is Dispensable for Adult Mouse Life
Source: PLoS One. 2015 Nov 16;10(11):e0143083. doi: 10.1371/journal.pone.0143083 (PMC4646642; doi:10.1371/journal.pone.0143083)
Supplement: S4 Fig — Representative images of tissue sections (H&E staining) from R26Cre-ERT2/Enpp2 n/n mice and littermates treated PO with Tmx (180 mg/kg) or corn oil for 6 days. Mice were sacrificed 18 and 30 days post Tmx administration. (Scale bar: 150 μm). (PDF) [file pone.0143083.s004.pdf]

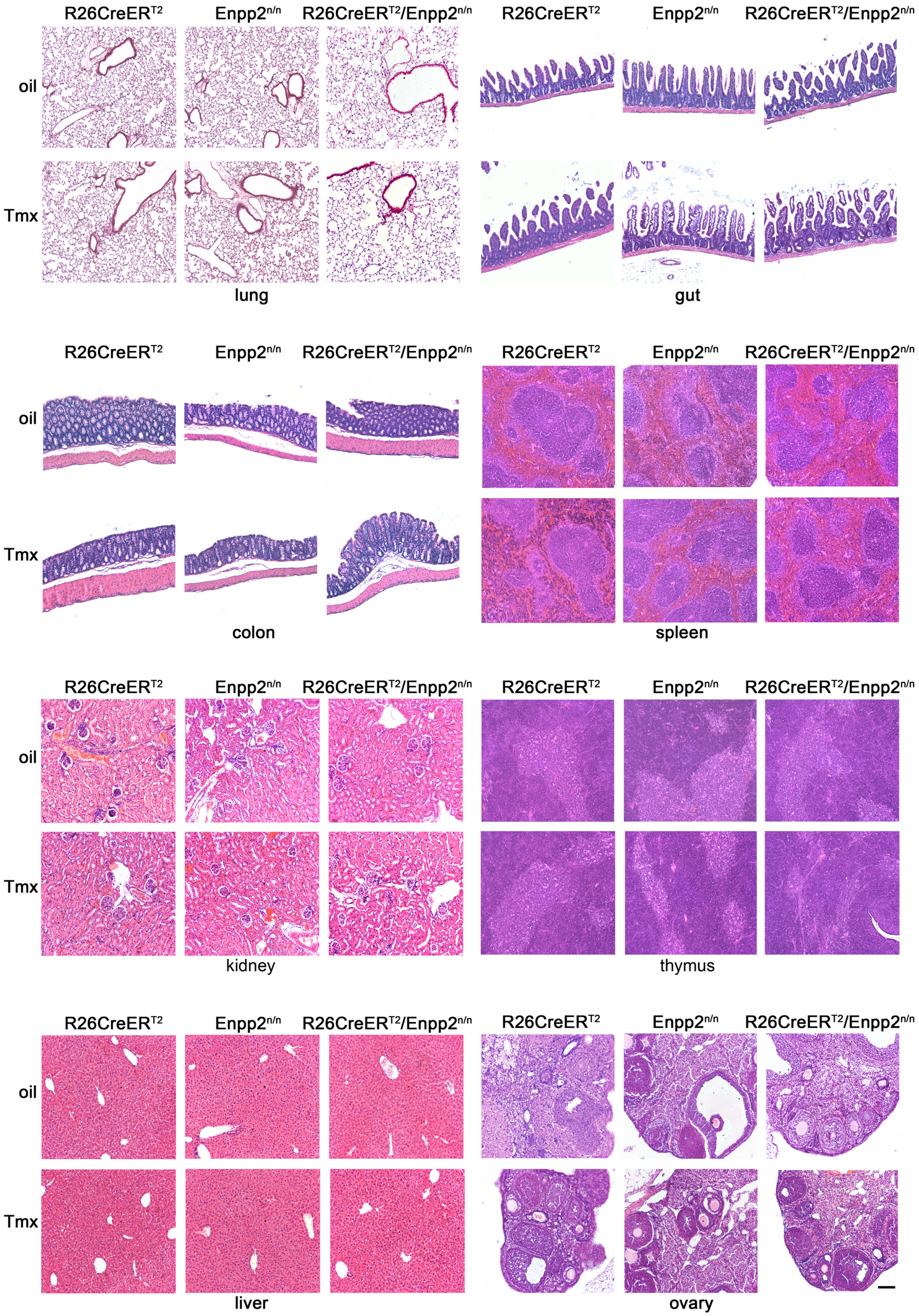

S4 Fig. Genetic deletion of *Enpp2* has no effect in tissue histology. Representative images of tissue sections (H&E staining) from R26Cre-ER^{T2}/Enpp2$^{n/n}$ mice and littermates treated PO with Tmx (180 mg/kg) or corn oil for 6 days. Mice were sacrificed 18 and 30 days post Tmx administration. (Scale bar: 150 μm).
