# Supplementary material for: The Bulk of Autotaxin Activity Is Dispensable for Adult Mouse Life
Source: PLoS One. 2015 Nov 16;10(11):e0143083. doi: 10.1371/journal.pone.0143083 (PMC4646642; doi:10.1371/journal.pone.0143083)
Supplement: S5 Fig — Plasma biochemical analytes from Tmx / oil-administered R26Cre-ERT2/Enpp2 n/n mice and littermates (n = 3–8, exp = 2). AST: Aspartate transaminase; ALT: Alanine transaminase; CPK: Creatine phosphokinase; LDH: Lactate dehydrogenase; γ-GT: γ-glutamyl-transpeptidase. (PDF) [file pone.0143083.s005.pdf]

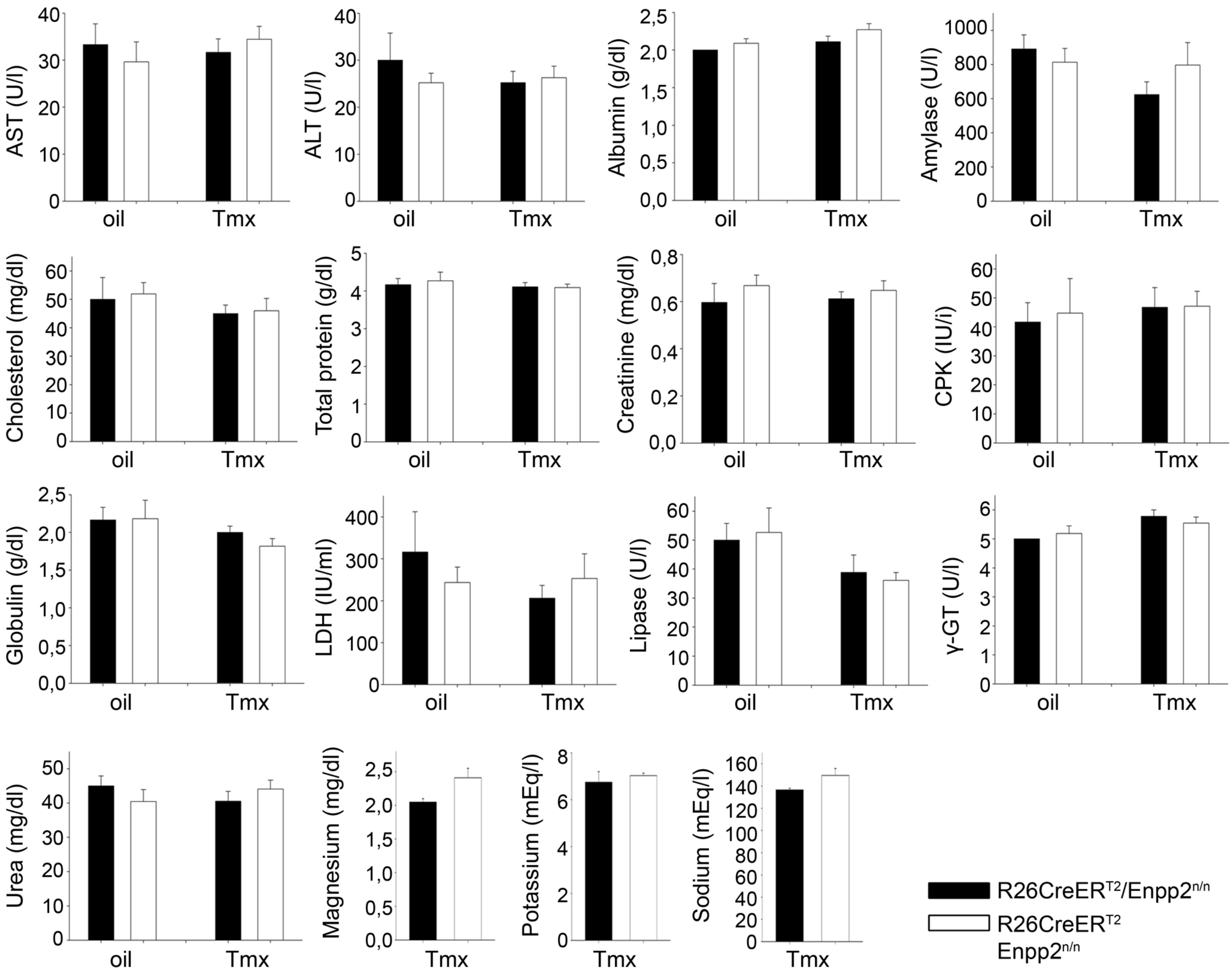

S5 Fig. Genetic excision of *Enpp2* has no effect in biochemical factors indicative of main body functions. Plasma biochemical analytes from Tmx / oil-administered R26Cre-ER^{T2}/Enpp2$^{n/n}$ mice and littermates (n=3-8, exp=2). AST: Aspartate transaminase; ALT: Alanine transaminase; CPK: Creatine phosphokinase; LDH: Lactate dehydrogenase; γ-GT: γ-glutamyl-transpeptidase.
