# Supplementary material for: The Bulk of Autotaxin Activity Is Dispensable for Adult Mouse Life
Source: PLoS One. 2015 Nov 16;10(11):e0143083. doi: 10.1371/journal.pone.0143083 (PMC4646642; doi:10.1371/journal.pone.0143083)
Supplement: S6 Fig — Blood cell counts and parameters in Tmx-treated R26Cre-ERT2/Enpp2 n/n mice and littermates (n = 3–6, exp = 1). WBC: White blood cells; LYMPH: Lymphocytes; MXD: monocytes, basophils and eosinophils; GRA: Granulocytes; RBC: Red blood cells; HGB: hemoglobin; HCT: hematocrit; MCV: mean volume of erythrocytes; MCH: mean content of hemoglobin; MCHC: mean concentration of hemoglobin; RDW: Red cell Distribution Width; PLT: platelets; PCT: plateletkrit; MPV: mean platelet volume; PDW: relative width of the distribution of platelets. (PDF) [file pone.0143083.s006.pdf]

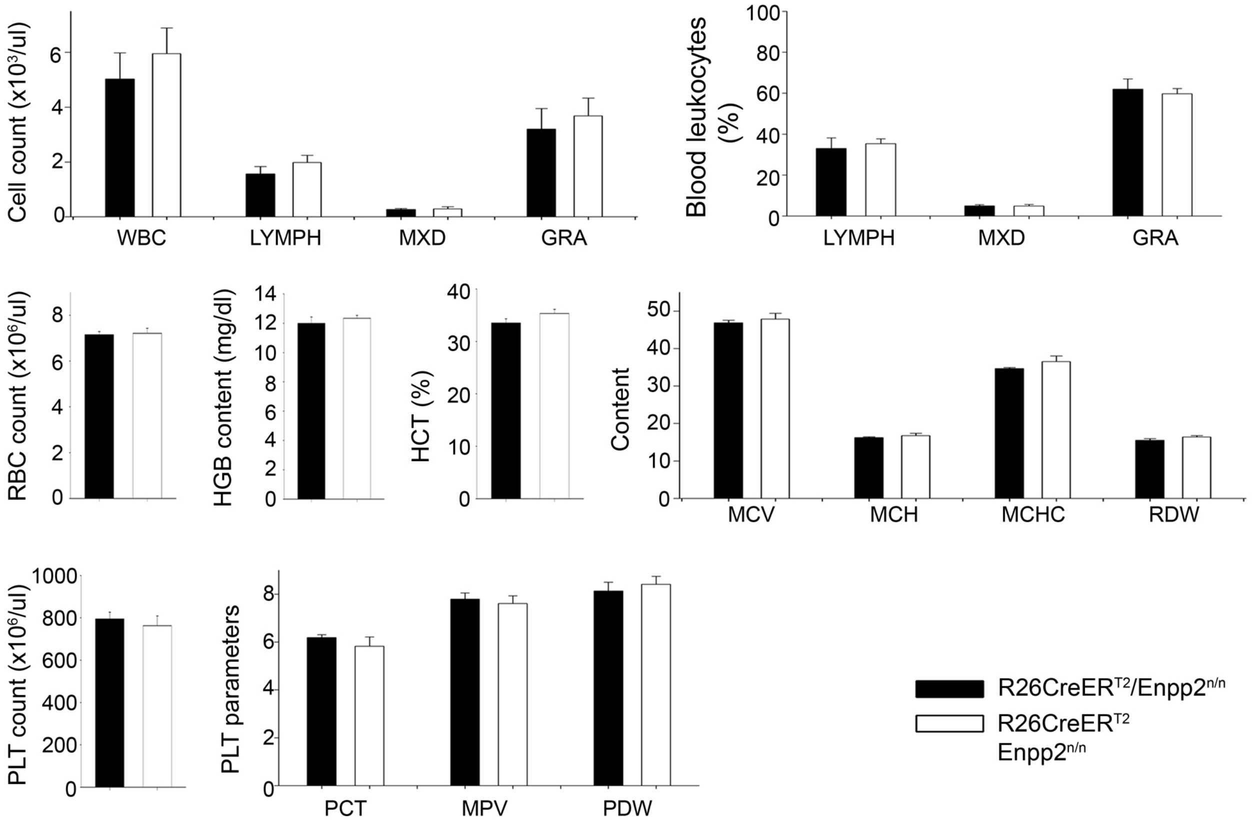

S6 Fig. Genetic excision of *Enpp2* has no effect in hematopoietic cell populations. Blood cell counts and parameters in Tmx-treated R26Cre-ER^{T2}/Enpp2$^{n/n}$ mice and littermates (n=3-6, exp=1). WBC: White blood cells; LYMPH: Lymphocytes; MXD: monocytes, basophils and eosinophils; GRA: Granulocytes; RBC: Red blood cells; HGB: hemoglobin; HCT: hematocrit; MCV: mean volume of erythrocytes; MCH: mean content of hemoglobin; MCHC: mean concentration of hemoglobin; RDW: Red cell Distribution Width; PLT: platelets; PCT: plateletcrit; MPV: mean platelet volume; PDW: relative width of the platelet distribution.
