# Supplementary material for: The Bulk of Autotaxin Activity Is Dispensable for Adult Mouse Life
Source: PLoS One. 2015 Nov 16;10(11):e0143083. doi: 10.1371/journal.pone.0143083 (PMC4646642; doi:10.1371/journal.pone.0143083)
Supplement: S7 Fig — (A) Western blot of the indicated mouse plasma samples with a rat monoclonal antibody (4F1) against ATX. (B) Western blot of the same (as in A) samples with a commercial (Cayman) rabbit polyclonal against ATX. (C) Coomassie brilliant blue staining of the same (as in A) samples, serving as a loading control. (PDF) [file pone.0143083.s007.pdf]

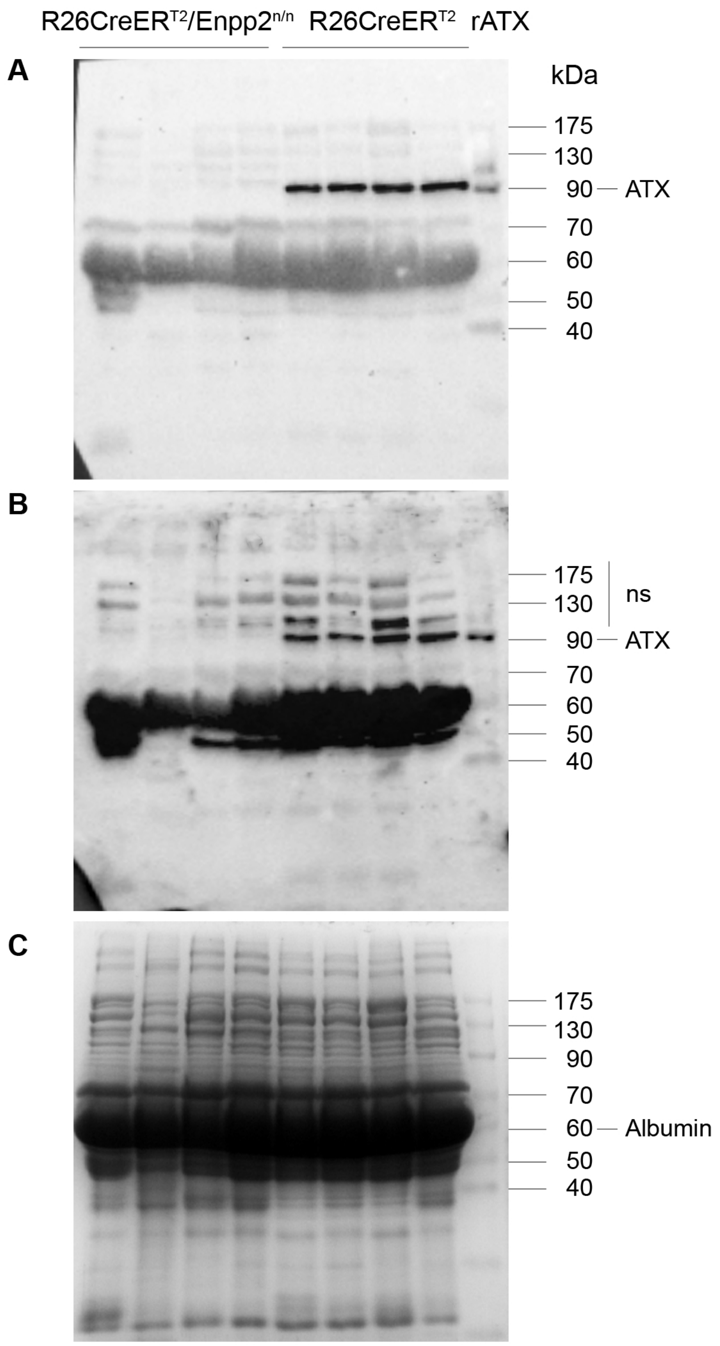

S7 Fig. Genetic excision of *Enpp2* attenuates its protein levels in the plasma. A. Western blot of the indicated mouse plasma samples with a rat monoclonal antibody (4F1) against ATX. **B.** Western blot of the same (as in A) samples with a commercial (Cayman) rabbit polyclonal antibody against ATX. **C.** Coomassie brilliant blue staining of the same (as in A) samples, serving as a loading control.
