# Supplementary material for: The Bulk of Autotaxin Activity Is Dispensable for Adult Mouse Life
Source: PLoS One. 2015 Nov 16;10(11):e0143083. doi: 10.1371/journal.pone.0143083 (PMC4646642; doi:10.1371/journal.pone.0143083)
Supplement: S8 Fig — Representative images of tissue sections from vehicle-treated and PF8380-treated mice (120 mg/kg PF8380, PO, twice a day for 3 weeks), stained with H&E. (Scale bar: 150 μm). (PDF) [file pone.0143083.s008.pdf]

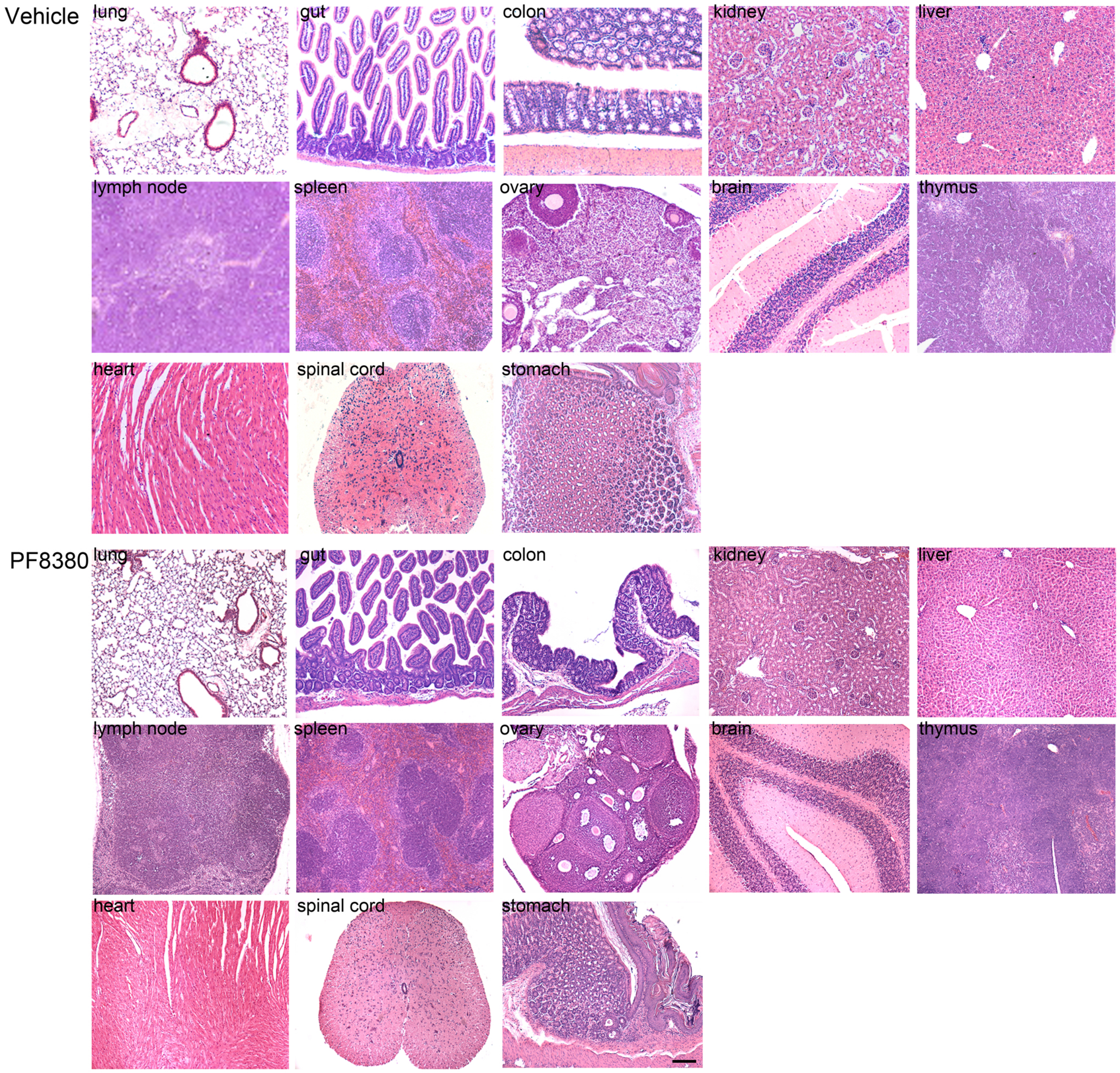

S8 Fig. Potent pharmacological inhibition of ATX has no effect on tissue histology. Representative images of tissue sections from vehicle-treated and PF8380-treated mice (120 mg/kg PF8380, PO, twice a day for 3 weeks), stained with H&E. (Scale bar: 150 μm).
