# Supplementary material for: The Bulk of Autotaxin Activity Is Dispensable for Adult Mouse Life
Source: PLoS One. 2015 Nov 16;10(11):e0143083. doi: 10.1371/journal.pone.0143083 (PMC4646642; doi:10.1371/journal.pone.0143083)
Supplement: S1 Table — (PDF) [file pone.0143083.s009.pdf]

S1 Table. R26CreER^{T2}-driven *Enpp2* recombination, as tested by PCR, in the indicated mouse strains and upon the indicated treatments

	**Genotype**	ATXiCre	ATXiCre	ATXiCre	ATX	ATX	ATXiCre	ATXiCre	ATX	ATX	iCre	iCre
	**Treatment**	Tmx	Tmx	Oil	Tmx	Oil	Tmx	Oil	Tmx	Oil	Tmx	Oil
	**mg/Kg**	50	100	-	100	-	100	-	100	-	100	-
	**Route**	IP	IP	IP	IP	IP	IP	IP	IP	IP	IP	IP
	**Days**	10	10	10	10	10	5	5	5	5	5	5
**Recombination in tissues**	Brain	- 1/1	+ 1/1	nd	nd	nd	+ 4/4	+ 1/1	- 3/3	- 1/1	- 2/2	- 2/2
	Heart	- 1/1	+ 1/1	nd	nd	nd	+ 2/2	+ 1/2	- 3/3	- 1/1	- 2/2	- 2/2
	Lung	- 1/1	+ 6/6	+ 2/2	- 4/4	- 2/2	+ 8/8	+ 4/5	- 6/6	- 4/4	- 6/6	- 5/5
	Gut	-1/1	+ 6/6	+ 2/2	- 4/4	- 2/2	+ 11/11	+ 7/7	- 6/6	- 4/4	- 6/6	- 5/5
	Spleen	- 1/1	+ 6/6	+ 2/2	- 4/4	- 2/2	+ 11/11	+ 7/7	- 6/6	- 4/4	- 6/6	- 5/5
	Thymus	- 1/1	+ 1/1	nd	nd	nd	+ 4/4	+ 2/2	- 3/3	- 1/1	- 2/2	- 2/2
	Spinal cord	- 1/1	+ 1/1	nd	nd	nd	+ 3/3	+ 2/2	-3/3	- 1/1	- 2/2	- 2/2
	Kidney	- 1/1	+ 1/1	nd	nd	nd	+ 4/4	+ 1/1	- 3/3	- 1/1	- 2/2	- 2/2
	Liver	- 1/1	+ 1/1	nd	nd	nd	+ 4/4	+ 2/2	- 3/3	- 1/1	- 2/2	- 2/2
	Ovary	- 1/1	+ 1/1	nd	nd	nd	+ 3/3	+ 1/2	- 2/2	- 1/1	- 2/2	- 2/2
	Testis	- 1/1	+ 1/1	nd	nd	nd	+ 1/1	nd	-1/1	- 1/1	- 2/2	- 2/2
	Lymph nodes	nd	nd	nd	nd	nd	+ 3/3	+ 1/1	- 3/3	- 1/1	- 2/2	- 2/2

	**Genotype**	ATXiCre	ATXiCre	ATX	ATX	iCre	iCre	ATXiCre*	ATXiCre*
	**Treatment**	Tmx	Oil	Tmx	Oil	Tmx	Oil	Tmx	Oil
	**mg/Kg**	180	-	180	-	180	-	180	-
	**Route**	PO	PO	PO	PO	PO	PO	PO	PO
	**Days**	6	6	6	6	6	6	6	6
**Recombination in tissues**	Brain	+ 9/9	+ 2/2	- 4/4	- 3/3	- 5/5	- 3/3	+2/2	-2/2
	Heart	+ 8/8	+ 2/2	- 3/3	- 2/2	- 5/5	- 3/3	nd	nd
	Lung	+ 9/9	+ 2/2	- 3/3	- 3/3	- 5/5	- 3/3	+1/1	-2/2
	Gut	+ 11/11	+ 2/2	- 4/4	- 3/3	- 5/5	- 3/3	+1/1	-2/2
	Spleen	+ 11/11	+ 2/2	- 4/4	- 3/3	- 4/4	- 3/3	nd	nd
	Stomach	+ 8/8	+ 2/2	- 3/3	- 3/3	- 5/5	- 3/3	nd	nd
	Spinal cord	+ 9/9	+ 2/2	- 4/4	- 3/3	- 5/5	- 3/3	nd	nd
	Kidney	+ 9/9	+ 2/2	- 4/4	- 3/3	- 5/5	- 3/3	nd	nd
	Liver	+ 11/11	+ 2/2	- 4/4	- 3/3	- 6/6	- 3/3	+1/1	-2/2
	Ovary	+1/1	nd	- 2/2	- 2/2	- 1/1	- 1/1	nd	nd
	Testis	+ 6/6	+ 2/2	- 1/1	- 1/1	- 1/1	- 1/1	nd	nd
	Lymph nodes	+ 10/10	+ 2/2	- 4/4	- 3/3	- 5/5	- 3/3	nd	nd

ATX: homozygous Enpp2$^{n/n}$; iCre: heterozygous R26CreER^{T2};

IP: intraperitoneal; PO: oral; Tmx: Tamoxifen;

+ denotes detection of complete removal of floxed exons along with the neo cassette, as described previously (PMID:20079728)

nd: not determined

+1/2: denotes recombination in 1 out of 2 mice tested.

*denotes separate housing of mice receiving Tmx
