# Supplementary material for: The Bulk of Autotaxin Activity Is Dispensable for Adult Mouse Life
Source: PLoS One. 2015 Nov 16;10(11):e0143083. doi: 10.1371/journal.pone.0143083 (PMC4646642; doi:10.1371/journal.pone.0143083)
Supplement: S2 Table — (PDF) [file pone.0143083.s010.pdf]

S2 Table. No major effects in tissue physiology upon inducible, complete genetic deletion of ATX

	Mouse No	Affected tissues	Diagnosis	Severity
**R26Cre-ER^{T2}/Enpp2$^{n/n}$ +Tmx**	N295	Lung<br>Small gut<br>Colon<br>Cecum	Interstitial pneumonia<br>Enteritis<br>Typhlitis<br>Adult helminthes	Moderate<br>Mild<br>Mild<br>Mild
	N1	Lung<br>Small gut<br>Colon<br>Cecum<br>Spleen<br>Stomach	Interstitial pneumonia<br>Enteritis<br>Typhlitis<br>Typhlitis<br>Histiocytosis<br>Gastritis	Mild<br>Mild<br>Moderate<br>Moderate<br><br>Mild
	N303	Lung<br>Small gut<br>Colon<br>Cecum<br>Spleen	Interstitial pneumonia<br>Enteritis<br>Typhlitis<br>Typhlitis<br>Histiocytosis	Moderate<br>Mild<br>Moderate<br>Moderate
	N305	Lung<br>Small gut<br>Colon<br><br>Cecum	Interstitial pneumonia<br>Enteritis<br>Typhlitis<br>Colitis<br>Typhlitis	Mild<br>Mild<br>Moderate<br>Mild<br>Moderate
	N308	Lung<br>Small gut<br>Colon	Interstitial pneumonia<br>Enteritis<br>Typhylitis<br>Colitis	Moderate<br>Mild<br>Moderate<br>Mild
**Enpp2$^{n/n}$ +Tmx**	N300	Lung<br>Small gut<br>Colon	Interstitial pneumonia<br>Enteritis<br>Colitis	Severe<br>Mild<br>Severe
	N301	Lung<br>Small gut<br>Colon<br>Stomach<br>Lymph node<br>Spleen	Interstitial pneumonia<br>Enteritis<br>Colitis<br>Gastritis<br>Hystiocytosis, neutrophilia<br>Neutrophilia	Severe<br>Mild<br>Severe<br>Mild
	N304	Lung<br>Small gut<br>Colon<br>Stomach<br>Spleen	Interstitial pneumonia<br>Enteritis<br>Typhylitis<br>Gastritis<br>Hystiocytosis	Mild<br>Moderate<br>Moderate<br>Moderate
	N306	Lung<br>Small gut<br>Colon	Interstitial pneumonia<br>Enteritis<br>Typhylitis	Mild<br>Mild<br>Moderate
	N307	Lung<br>Small gut<br>Colon<br><br>Stomach	Interstitial pneumonia<br>Enteritis<br>Typhylitis<br>Colitis<br>Gastritis	Mild<br>Moderate<br>Severe<br>Severe<br>Moderate
**R26Cre-ER^{T2} +Tmx**	N339	Lung<br>Small gut<br>Colon	Interstitial pneumonia<br>Enteritis<br>Typhylitis<br>Colitis	Mild<br>Moderate<br>Severe<br>Severe
	N341	Lung<br>Small gut<br>Colon	Interstitial pneumonia<br>Enteritis<br>Typhylitis<br>Colitis	Mild<br>Moderate<br>Severe<br>Severe
	N342	Lung<br>Colon	Interstitial pneumonia<br>Typhylitis	Mild<br>Severe

			Colitis	Severe
	N343	Lung<br>Colon	Interstitial pneumonia<br>Typhylitis<br>Colitis	Mild<br>Severe<br>Severe
	N346	Lung<br>Colon	Interstitial pneumonia<br>Typhylitis<br>Colitis	Mild<br>Severe<br>Severe
	N250	Lung<br>Colon	Interstitial pneumonia<br>Typhylitis<br>Colitis	Mild<br>Severe<br>Severe
**R26Cre-ER^{T2} -Tmx**	N355	Lung<br>Small gut<br>Colon<br>Spleen	No significant findings<br>No significant findings<br>No significant findings<br>No significant findings	
	N357	Lung<br>Small gut<br>Colon<br>Spleen	No significant findings<br>Enteritis<br>No significant findings<br>No significant findings	Mild

Examined organs as arranged and phenotyped in slides: Slide 1: Heart, thymus, tongue, sternum; Slide 2: Lung, thyroid, trachea, salivary glands; Slide 3: Kidneys, adrenals; Slide 4: Liver, spleen, pancreas; Slide 5: GI tract; Slide 6: Reproductive organs, urinary bladder, skin (+mammary glands for females); Slide 7: Head (5 pieces); Slide 8: Hind limb; Slide 9: Vertebrae and spinal cord (cervical, thoracic, lumbar)
